# Morphological and molecular features of early regeneration in the marine annelid *Ophryotrocha xiamen*

**DOI:** 10.1038/s41598-022-04870-3

**Published:** 2022-02-02

**Authors:** Ruanni Chen, Irum Mukhtar, Shurong Wei, Siyi Wu, Jianming Chen

**Affiliations:** Institute of Oceanography, Minjiang University, Fuzhou, 350108 Fujian China

**Keywords:** Morphogenesis, Transcriptomics, Animal physiology, Developmental biology

## Abstract

Regeneration capability varies in the phylum Annelida making them an excellent group to investigate the differences between closely related organisms. Several studies have described the process of regeneration, while the underlying molecular mechanism remains unclear, especially during the early stage (wound healing and blastema formation). In this study, the newly identified *Ophryotrocha xiamen* was used to explore the early regeneration. The detailed morphological and molecular analyses positioned *O. xiamen* within ‘labronica’ clade. We analyzed the morphological changes during regeneration process (0–3 days post amputation) and molecular changes during the early regeneration stage (1 day post amputation). Wound healing was achieved within one day and a blastema formed one day later. A total of 243 DEGs were mainly involved in metabolism and signal transduction. Currently known regeneration-related genes were identified in *O. xiamen* which could help with exploring the functions of genes involved in regeneration processes. According to their conserved motif, we identified 8 different *Hox* gene fragments and *Hox5* and *Lox2* were found to be absent in early regeneration and during regular growth. Our data can promote further use of *O. xiamen* which can be used as an experimental model for resolving crucial problems of developmental biology in marine invertebrates.

## Introduction

Annelids are an excellent group to investigate regeneration abilities, as they exhibit various regeneration capacities^1–4^. The anterior and posterior regeneration are proved to be ancestral for Annelida^5^. Numerous species, including *Enchytraeus japonensis*, *Pristina leidyi* and *Cirratulus cirratus*, and *Eurythoe complanata*, have been reported to regenerate both anterior and posterior segments to form an entire individual^3,6–9^. Posterior regeneration is more widespread than anterior regeneration. Others, such as *Alitta virens* and *Capitella teleta* and species in *Ophryotrocha*, can regenerate segments posteriorly but not anteriorly ^4,10,11^. Although several studies describing the process of regeneration in both anterior and posterior regeneration annelids are available, investigating the molecular basis of species with the posterior regeneration ability only can help to distinguish the differences between anterior and posterior regenerative mechanisms in annelids.

For annelids, epimorphosis and morphallaxis are two different processes leading to regeneration. Early regeneration, mainly including wound healing and formation of blastema, involves muscular contraction and tissue autolysis at the site of the wound in annelids^12^. Several types of migrating cells are involved in early regeneration. Some of them are thought to phagocytize damaged tissues, while others help to regenerate new tissues. Previous studies have mainly focused on morphological changes and cell division, while comparative transcriptome analysis can help in understanding the regeneration process^6–8,10^. Numbers of genes, including *Hox* genes and several genes of the germline multipotency program (GMP), are demonstrated to be involved in early regeneration in annelids^10,13–17^.

Annelids of the genus *Ophryotrocha* Claparède & Mecznikow, 1869 (Errantia: Dorvilleidae) are distributed in a wide range of habitats from shallow water to the deep sea^18–20^. To date, approximately 86 species in this genus have been described according to the GBIF data but records from the coastal zones of China are scarce^21^. Molecular characters support four clades within *Ophryotrocha*, the ‘labronica’, ‘hartmanni’, ‘lobifera’, and an undefined clade, identified with 16S, *cytochrome c oxidase I* (*COI*), and *histone 3* (*H3*) sequences^22,23^.

Due to their capability of laboratory maintenance, high fecundity, short generation time, and rapid individual growth rate, some species of *Ophryotrocha* have been used as model organisms of marine invertebrates in the fields of genetics, reproduction, development, and regeneration^19,24^. Morphological descriptions illustrate that both *Ophryotrocha puerilis* and *O. notoglandulata* regenerate posteriorly and no anterior segment regeneration occurs when only part of the prostomium remains^4,25^. The molecular mechanisms may provide new features of regeneration in annelids, however, no molecular data describing regeneration in *Ophryotrocha* have been available until now.

In this study, we reported a new species of *Ophryotrocha*, *Ophryotrocha xiamen* **sp. nov.**. The morphological characteristics, the phylogenetic evidence using mitochondrial (*COI*) and nuclear (*H3*) markers and the ultrastructural changes after amputation were studied. We used RNA sequencing (RNA-seq) followed by de novo transcriptome assembly to characterize early regeneration in *O. xiamen* for the first time. Furthermore, we analyzed the difference between regular growth and regeneration after amputation at the transcriptome level and documented regeneration-related genes to reveal unique features in early regeneration. The present study reported a new species of *Ophryotrocha* and provided new insights into the early regeneration process at the morphological and molecular levels.

## Material and methods

### Culture and life cycle of *O. xiamen*

**Figure 1:**
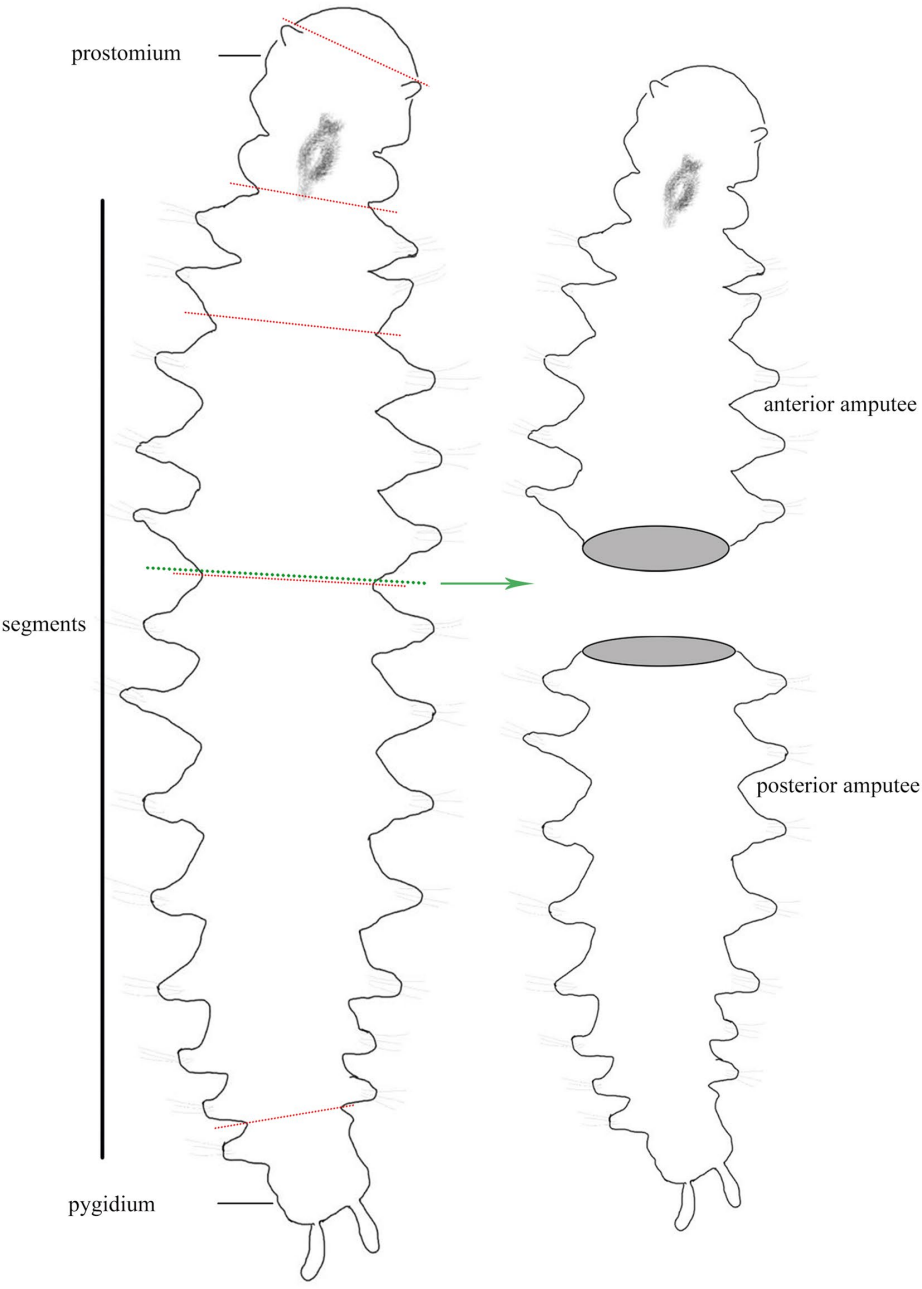
Illustration of the amputation levels in *Ophryotrocha xiamen* **sp. nov.**. Dashed line represents the dissection region for morphological (red) and transcriptome (green) experiment. Both anterior and posterior amputees were fixed for transcriptome sequencing (see Methods). The figure was created with Photoshop CS6.

*Ophryotrocha xiamen* worms were collected from Baicheng Bay, Xiamen, China (118.08E, 24.44 N), and cultured in our laboratory for more than two years. The worms were kept in Petri dishes with sterilized seawater and fed with an artificial diet (shrimp diet, Fuxing Feed Co., Ltd. Xiamen, China). The salinity, water temperature, and photoperiod were maintained at 23–25‰, 24 ± 1 °C and 12 light: 12 dark, respectively. For regeneration experiments, approximately 19–26 days-old adult specimens with 12–15 segments, were anesthetized in 10% MgCl_2_ solution in artificial seawater and dissected at the prostomium, 0, 2–4, 6–8, and 10–12 post-pharynx segments using scalpels, respectively (Fig. 1). Both anterior and posterior amputees were placed in Petri dishes, each containing artificial seawater.

### Karyotype analysis

Chromosome preparations were obtained from worms with 3–10 segments. The living specimens were incubated in a seawater colchicine solution (0.05%) for 22–24 h at 24 ± 1 °C, then subjected to a hypotonic solution (0.075 mol/L Kcl) for 60 min. Next, the worms were fixed with freshly prepared 3:1 Methanol: Acetic Acid fixative, with three fixative changes for 30 min at 4 ℃. Five to eight worms were transferred to the same clean slide and gently squashed with glass needles, air-dried, and stained with 10% Giemsa stain in phosphate-buffered saline (ph = 6.8) for 15 min. after that, the samples were gently squashed under a coverslip. Eventually, the slides were examined under a ZEISS Axioscope 5 microscope (Carl Zeiss AG, Oberkochen, Germany).

### DNA extraction

Cultures were kept without feeding for 3 days to ensure the purity of the samples and were rinsed 3 times with sterilized seawater containing penicillin G (100 U/ml) and streptomycin (100 mg/ml). DNA was extracted using a TIANamp Marine Animals DNA Kit (DP324, Tiangen Biotech Co. Ltd., Beijing, China) according to the manufacturer's instructions. The COI^26^ and H3 genes^27^ were amplified using the primers listed in Table 1. Amplification cycle conditions were as follows: 5 min at 94 °C, followed by 32 cycles at 94 °C for 30 s, 51 °C (COI)/56 °C (H3) for 30 s, 72 °C for 30 s, and a final extension at 72 °C for 10 min. The PCR products were sequenced with the amplification primers by Shanghai Shenggong Co., Ltd. (Shanghai, China).

**Table 1:** Sets of primers used to amplify COI and H3 in this study.

Primers	Sequences (5'-3')	References
COI F	GGTCAACAAATCATAAAGATATTGG	
COI R	TAAACTTCAGGGTGACCAAAAAATCA	Folmer et al.^26^
H3 F	ATGGCTCGTACCAAGCAGACVGC	
H3 R	ATATCCTTRGGCATRATRGTGAC	Colgan et al.^27^

### Phylogenetic analysis

Molecular phylogenetic analyses were performed with data sets from the sequences *COI* and *H3*. In total, 43 terminal taxa were used in the analyses, including *O. xiamen*, 37 other species from the genus *Ophryotrocha*, 5 species from other genera within Dorvilleidae, and rooted using *Eunice pennata* (Supplementary Table S1). The aligned sequences were used as data sets to generate the genetic distance using Kimura’s two-parameter (K2P) model. Based on the K2P distances, we calculated the interspecific genetic differences among the closest taxa. The phylogenetic trees were constructed by the maximum likelihood method (ML) using MEGA software (version 5.05), with 1,000 bootstrap pseudo replicates. For the ML analysis, the molecular datasets were combined and run in jModelTest with BIC, which suggested GTR + I + G as the best model.

### RNA extraction, library construction and Illumina sequencing

For RNA extraction, about 60 individuals with 12–15 segments were amputated in the midbody (6–8 post-pharynx segments; Fig. 1). Both anterior and posterior parts were merged and collected 1 day after amputation. The control group (60 individuals) without amputation was cultured under the same conditions as the regeneration group. Total RNA was extracted using MiniBEST Universal RNA Extraction Kit (TaKaRa, 9767) according to the manufacturer’s instructions. The integrity and size distribution of two RNA samples were verified using an Agilent 2100 Bioanalyzer (Agilent Technologies, CA, USA). Total RNA with RNA integrity numbers (RINs) ≥ 7.5 was used for cDNA library preparation. The mRNA-seq libraries were performed at Beijing Berry Genomics Co., Ltd. (Beijing, China). The mRNA was purified from the total RNA using poly-T oligo-attached magnetic beads. The cleaved RNA fragments were reverse transcribed into first-strand cDNA using random primers and then synthesized into double-stranded cDNA. From the cDNA, paired-end libraries were synthesized for both control and 1 day post amputation. Short fragments were purified with the QIA quick PCR Purification Kit (Qiagen), which was also used for continued end repair and ‘A’ base addition. These fragments were then ligated to adapters and purified through gel separation. Finally, the adaptor-ligated libraries were amplified by PCR for sequencing. Illumina sequencing was performed using the NovaSeq 6000 platform, and 150 bp paired-end reads were generated. Raw sequences were deposited in the NCBI Short Read Archive (SRA) database (http://www.ncbi.nlm.nih.gov/Traces/sra/) under accession numbers: SRR12074689 and SRR12074688.

### Assembly sequencing and functional annotation

After removing the adaptor sequences, ambiguous ‘N’ nucleotides (with the ratio of ‘N’ greater than 10%) and low-quality sequences (with a quality score of less than 5) using Trimmomatic, the remaining clean reads were assembled using Trinity for transcriptome assembly without a reference genome. The longest transcript of each single gene was selected as a unigene. For annotation analysis, unigenes were BLASTX-searched against seven databases, including the National Center for Biotechnology Information (NCBI) nonredundant protein sequence (Nr) database, nonredundant nucleotide sequence (Nt) database, Protein family (Pfam), Clusters of Orthologous Groups (KOG/COG), Gene Ontology (GO), Kyoto Encyclopedia of Genes and Genomes (KEGG) Orthology (KO) database (www.kegg.jp/kegg/kegg1.html)^28^, and the Swiss-Prot, using a cutoff E-value of 10–5. Differentially expressed genes (DEGs) between control and regeneration groups (1 day post amputation) were identified with DEGseq analysis from the adjusted read count data^29^. The Benjamini & Hochberg method was used to adjust the *P*-values. Significantly differential expressed genes were determined by setting the threshold of corrected *P*-value of 0.05 and log2 (Fold change) of 1. Unigenes were annotated based on the BLASTX results, and the best alignments were used for downstream analyses. GO and KEGG databases were both used to predict the functions of unigenes.

### Analysis of the Hox genes during the early regeneration

*Hox* genes have been implicated in wound healing and the dedifferentiation process during the early stage of regeneration^10,13–15^. We performed a local BlastP search against the predicted amino acid database with an E-value cutoff at 1e−3 by using the highly conserved homeodomain (60 amino acid residues) and ten flanking positions of the Hox protein. We used published sequences that were only full or near full length accessible from the GenBank database at NCBI (Supplementary Table S2). As mentioned above (Phylogenetic analysis section), the alignments of protein sequences were generated using ClustalW with the BLOSUM matrix, gap-opening penalty of 10 and gap-extension penalty of 0.1. The datasets for ML analysis were combined and run in the Jones Taylor Thornton (JTT) model.

### Scanning electron microscopy (SEM) for the morphology of *O. xiamen*

The morphological characters of whole worms, anterior and posterior parts were investigated by using SEM, and the preparation was conducted according to a previous method^30^. Living specimens were washed thrice using sterilized seawater and suspended in 2.5% glutaraldehyde at 4 °C for 24 h and then transferred to a mixture of a saturated solution of HgCl2 and 1% OsO4 (4:1) at 4 °C for 10 min. All solutions mentioned above were diluted in sodium cacodylate buffer (pH 7.2) followed by specimen dehydration in a graded ethanol series, critical point drying, setting on aluminum stubs, and sputter-coating with platinum. The prepared samples were examined with a JSM-6380LV SEM (JEOL, Tokyo, Japan) at the Fujian Academy of Agricultural Sciences.

## Results

### Systematics

*Ophryotrocha xiamen* **sp. nov.**

Dorvilleidae Chamberlin, 1919.

*Ophryotrocha* Claparède & Mecznikow, 1869.

Material examined. Taiwan Strait, Baicheng bay (China), 24.44′N, 118.08′E, 0–3 m depth. The type isolated by designation is F3-7, deposited at the Institute of Oceanography, Minjiang University.

**Figure 2:**
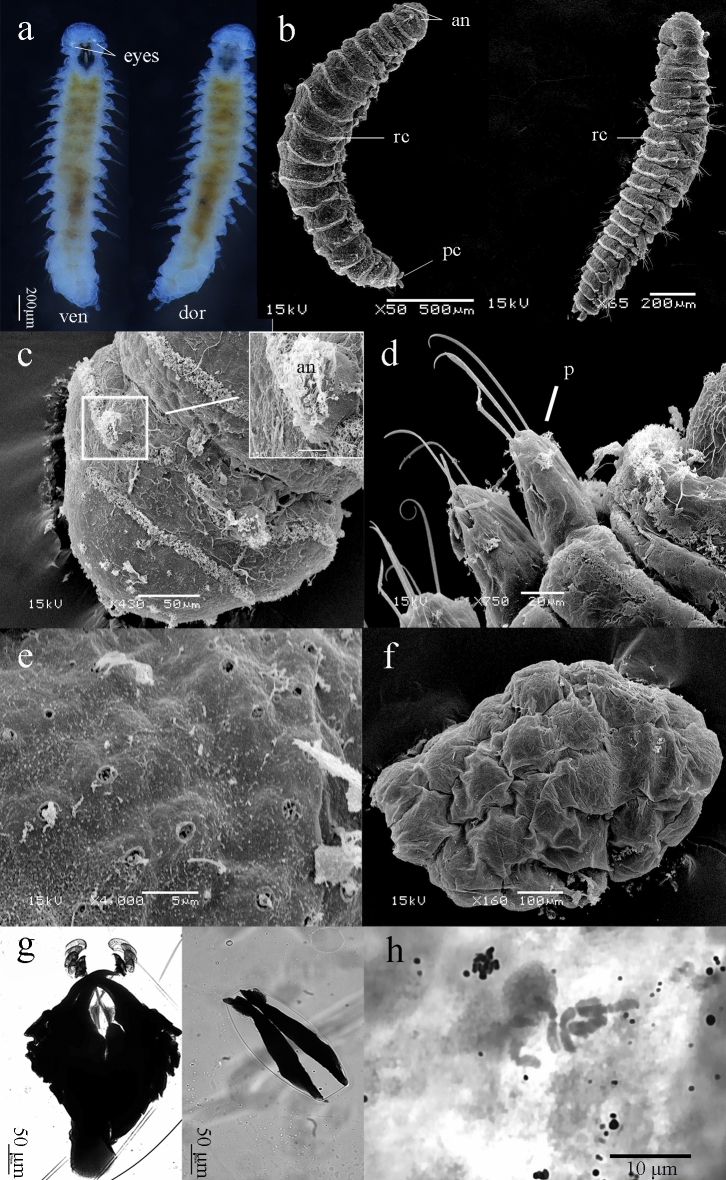
*Ophryotrocha xiamen*. (**a**) Living adult; (**b**) SEM image of *O. xiamen*; (**c**) Head region with paired antennae; (**d**) Median parapodia; (**e**) Image of rosette gland; (**f**) Egg-cocoon; (**g**) K-maxillae and mandible of 17-chaetigers; (**h**) Chromosomes of *O. xiamen* (2n = 6). an: antennae; dor: dorsal view; pc: pygidial cirri; rc: rings of cilia; ven: ventral view. Scale bars showed at the bottom of each image. The lights microscope images were taken using Zen2.6 Blue edition software.

Description. The Specimens (n = 15) with 24 segments (maximum number of segments) measured 3.10 ± 0.44 mm. Body shape dorso-ventrally flattened, narrow, tapering gently to pygidium, color opaque white in alcohol (Fig. 2a,b). Jaws and paired light-reflecting eyes in the enlarged head could be observed under microscope (Fig. 2a). The head displaying paired digitiform antennae surmounted with the curved cilia was similar to *O. labronica* and *O. japonica* (Fig. 2c). Dorsal and ventral bundles of cilia were present throughout the body, interrupted by parapodia (Fig. 2d). The pygidium was observed bearing two pygidial cirri and a median stylus. The median stylus only appeared in larval stage and disappeared in adult stage (Fig. 2b). Rosette glands (Fig. 2e), one per segment, presented mid-dorsally on the posteriormost segments of the mature animals. Glands appeared in adults of 10 to 12 chaetigers. The life history events are given in Supplementary Table S3. Tube-shaped egg-cocoons (Fig. 2f, Supplementary Fig. S1) were found protected by the female and each cocoon contained approximately 150–230 zygotes. Seven days after egg-laying, larvae with a short pygidial stylus were released from the cocoon as two-segmented individuals at 25 °C and further growth was achieved by adding new segments before the pygidium. They moved around the bottom or the seawater surface using both parapodia and rings of cilia on their surface. We also notice that the adults can produce a network of mucous trails that may be recognized by conspecifics. The mandibles showed no significant changes during the life-cycle while the maxillae in worms with 12 segments started to change to K-type maxillae (Fig. 2g). The molting time was different between males and females, the change occurred early in males. Many oocytes were concentrated in the middle and posterior regions of the coelom. The first spawning was observed at 28 days.

Reproduction and development. Gonochoristic; chromosomes 2n = 6 (Fig. 2h); mean diameter of eggs 93 ± 22 μm, released larvae with 2–3 segments, with a short pygidial median stylus.

Etymology. *Ophryotrocha xiamen* **sp. nov.** was first discovered in Xiamen, hence the name.

**Figure 3:**
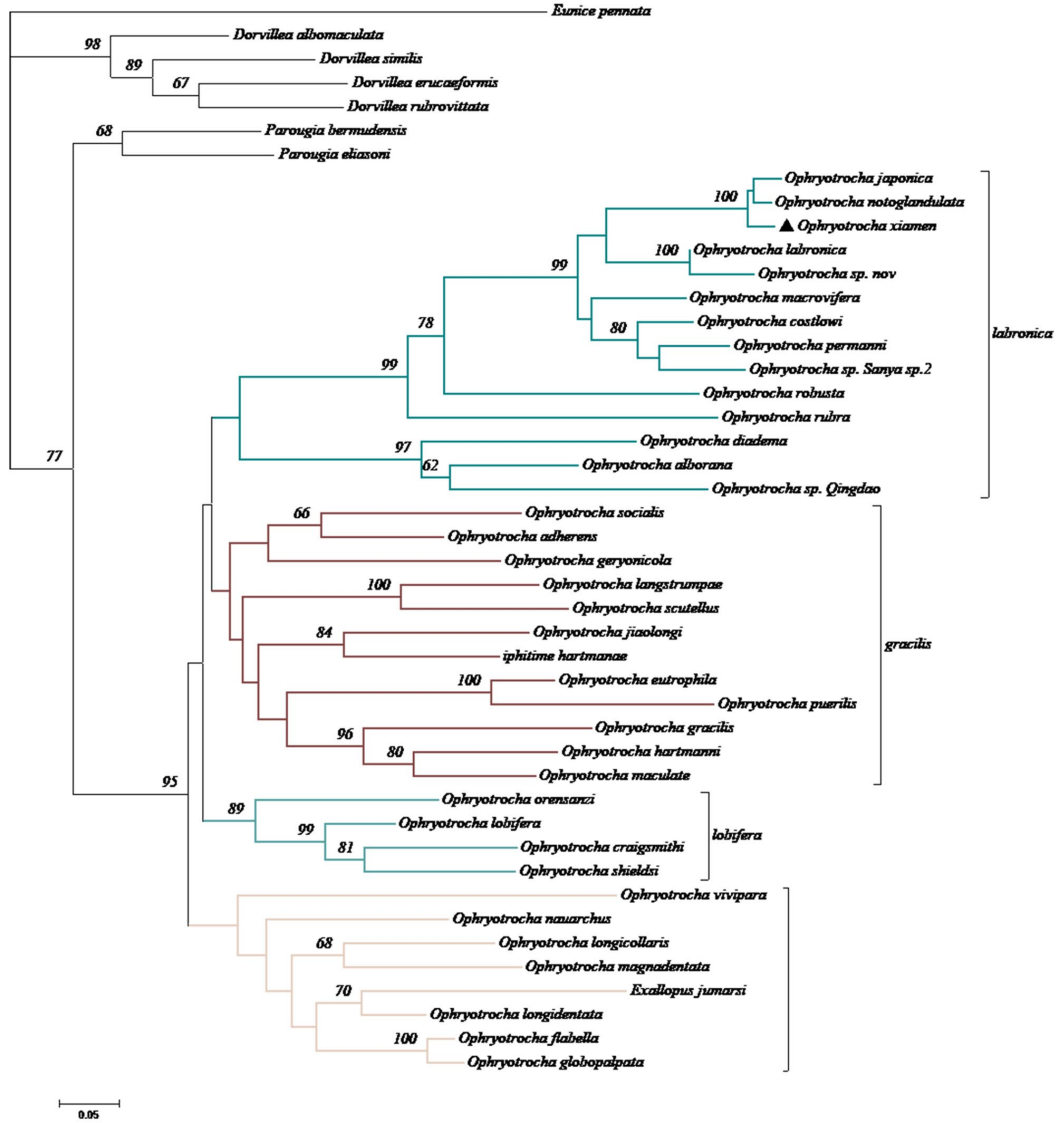
Phylogenetic analyses of a combined data set with two genes (COI and H3) using MEGA 5.05. The tree was calculated using the maximum parsimony method (bootstrap 1000 replications). The scale bar indicates 0.05 nucleotide substitutions per site. A symbol ▲indicate *O. xiamen* from this study.

Phylogenetic analysis. The combined alignment consisted of 804 bp, of which COI had 524 bp and H3 had 280 bp. Phylogenetic analyses resulted in similar tree topologies regardless of which tree reconstruction methods were used, therefore, only the results from the maximum likelihood analysis were discussed and shown (Fig. 3). *O. xiamen* fell within the ‘labronica’ clade. Genetic distance analyses showed that interspecific sequence divergence ranged from 5.93–30.70% for COI and 2.69–6.85% for H3.

### Morphological characterization of early regeneration events

**Figure 4:**
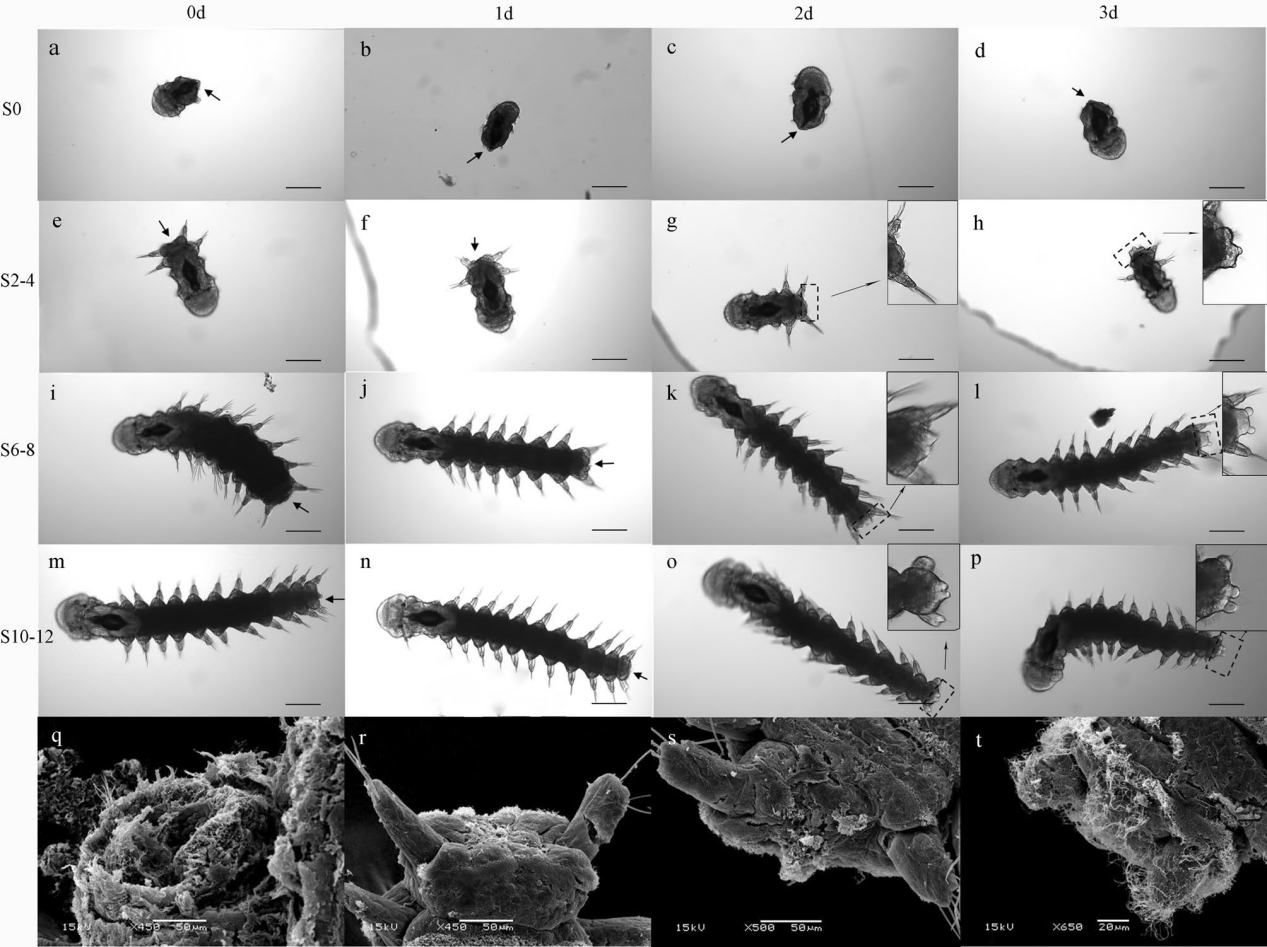
Morphological characterizations of the early posterior regeneration in *O. xiamen* during 3 days post amputation. (**a**–**d**) Worms amputated at post-pharynx segments 0 (S0); (**e**–**h**) Worms amputated at post-pharynx segments 2–4 (S2-4); (**i**–**l**) Worms amputated at post-pharynx segments 6–8 (S6-8); (**m**–**p**) Worms amputated at post-pharynx segments 10–12 (S10-12); (**q**–**t**) SEM images of S6-8 during 3 days post amputation. After 3 days post amputation, a pygidium bearing two short cirri was restored. All pictures showed in dorsal view. The arrows and dotted boxes show dissection/regeneration regions. Scale bars 0.2 mm. The lights microscope images were taken using Zen2.6 Blue edition software.

The anterior and posterior regeneration abilities were investigated using worms with 12–15 segments. A rapid regeneration process was observed in posterior growth (Fig. 4), in contrast, anterior growth was a slow process. The internal organs and coelomic fluid were oozed out immediately after amputation (Fig. 4a,e,i,m,o). In general, the morphological description of the posterior regeneration stages (n = 30) were as follows: day 1, after amputation, the edges of the cut gut fused with the edges of the cut body wall, reforming a posterior opening. This process was similar to typical healing by fusion with tissues, indicating that wound healing was already achieved (Fig. 4b,f,j,n,r); day 2, a posterior protuberance had already been observed indicating that blastema formation might have started at day 1 (Fig. 4c,g,k,o,s); and day 3, as the regeneration proceeded, the growing protuberance increased in size, and a complete pygidium bearing two pygidial cirri could be restored (Fig. 4d,h,l,p,t).

Worms were amputated at post-pharynx segments 0, 2–4, 6–8, and 10–12 to assess the influence of different amputation sites. During the first 3 days after amputation, the worms, except those amputating at post-pharynx segment 0, were able to form a complete regenerate pygidium with two pygidial cirri (Fig. 4t). The survival rates of different position of the amputation plane were showed in Supplementary Fig. S2.

Interestingly, we observed that *O. xiamen* could only partially restore the anterior end, but no new segment was observed. This happened only when part of the prostomium remained. The anterior regeneration process was completed within two weeks (Supplementary Fig. S3). Although worms without a peristomium could survive for months, they eventually starved to death.

### Transcriptome overview

In total, 16.02 and 18.52 million raw reads were generated from Ox t (1 day post amputation) and Ox c (uncut control) of *O. xiamen*, respectively. After removal of the adaptor sequences, ambiguous reads and low-quality reads, a total of 23,712,145 reads comprising 7,099,167,646 bases from the control group and 24,034,889 reads comprising 7,195,652,874 bases from the regeneration group (1 day post amputation) were obtained. In total, 72,980 unigenes were obtained with a mean length of 1180 bp, 41.1% GC content and an N50 of 1,976 bp. Based on the size distribution analysis, the lengths of 25,613 unigenes (35.1%) were > 1000 bp (Table 2).

**Table 2:** Quality parameters of illumine transcriptome sequencing of *O. xiamen*.

*Data generation and filtering*	
Total number	72,980
Total length	86,140,670
GC content (%)	49.92

*Assembly statistics*	
300–1000 (bp)	38,088 (44. 28%)
1000–3000 (bp)	19,941 (23.18%)
> 3000 (bp)	9,970 (11.59%)
Unigenes	86,017
Total length (bp)	83,647,650
N50 length (bp)	1,505
Mean length (bp)	972.41

**Figure 5:**
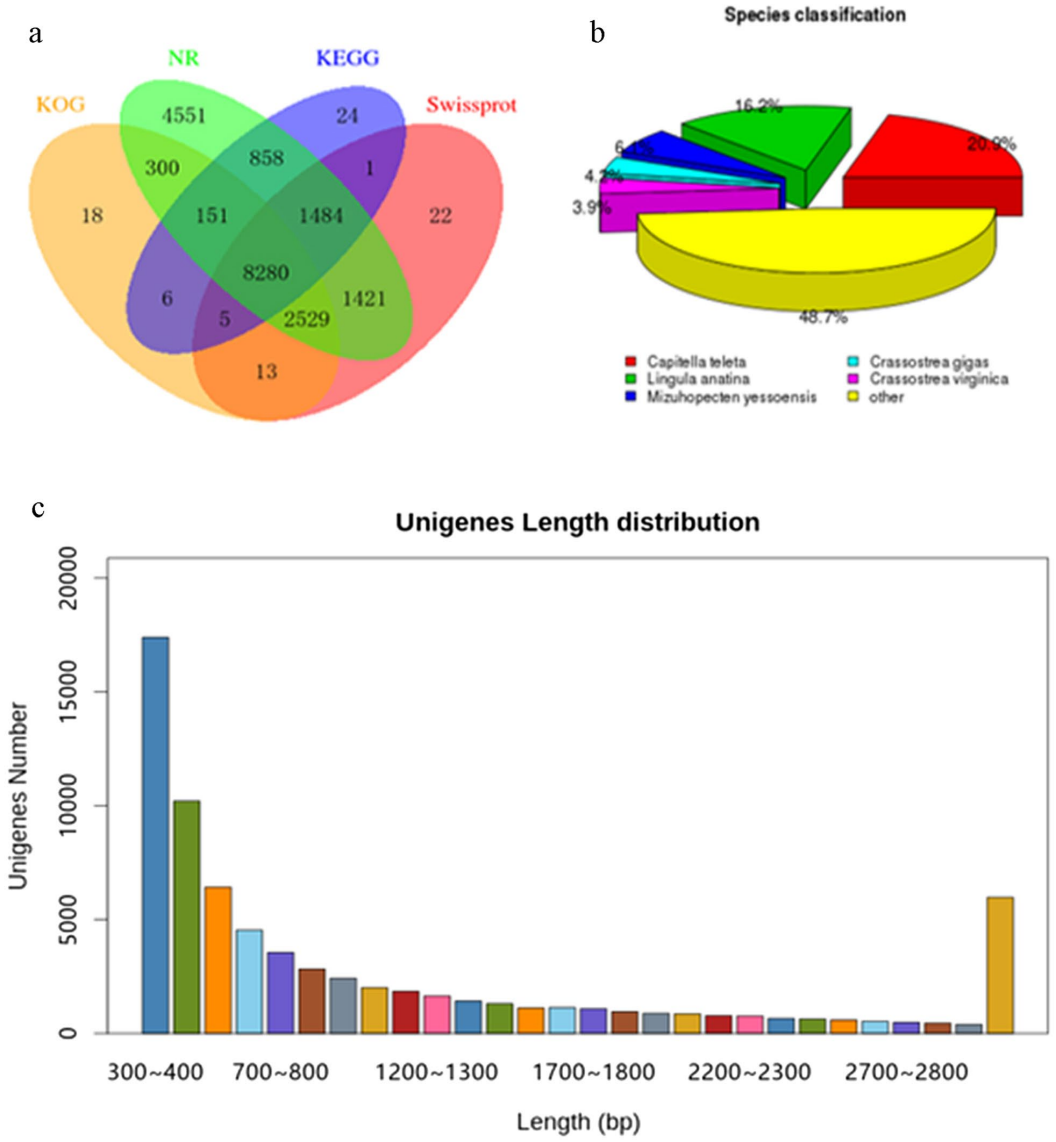
Length distribution and gene annotation in the transcriptional profile. (**a**) Venn diagram of BLAST hits for unigenes against protein databases (E-value < 1e−05). (**b**) Species classification of BLAST hits for the assembled unigenes. (**c**) Length distribution of the assembled unigenes.

Among them, 19,574, 3048, and 13,755 unigenes were matched in the NR, NT, and SwissProt databases, respectively. More than 62.5% of the unigenes possessed an E-value of more than 1e−30. Among the database proteins that matched predicted proteins, *O. xiamen* unigenes had the highest number of hits to *Capitella teleta* (20.9%), a polychaete worm, followed by *Lingula anatina* (16.2%) (Fig. 5).

For functional predictions and categories, all 8788 unigenes were assigned to three functional GO terms, including cellular component (452 subcategories), molecular function (180 subcategories), and biological process categories (2114 subcategories). Cell (4100 unigenes) and cell part (3958 unigenes) were the main subcategories in the cellular component category, while the main subcategories in molecular function were catalytic activity (3344 unigenes) and binding (3271 unigenes), and the major biological process was cellular process (4944 unigenes).

### Comparative gene expression during regeneration

**Figure 6:**
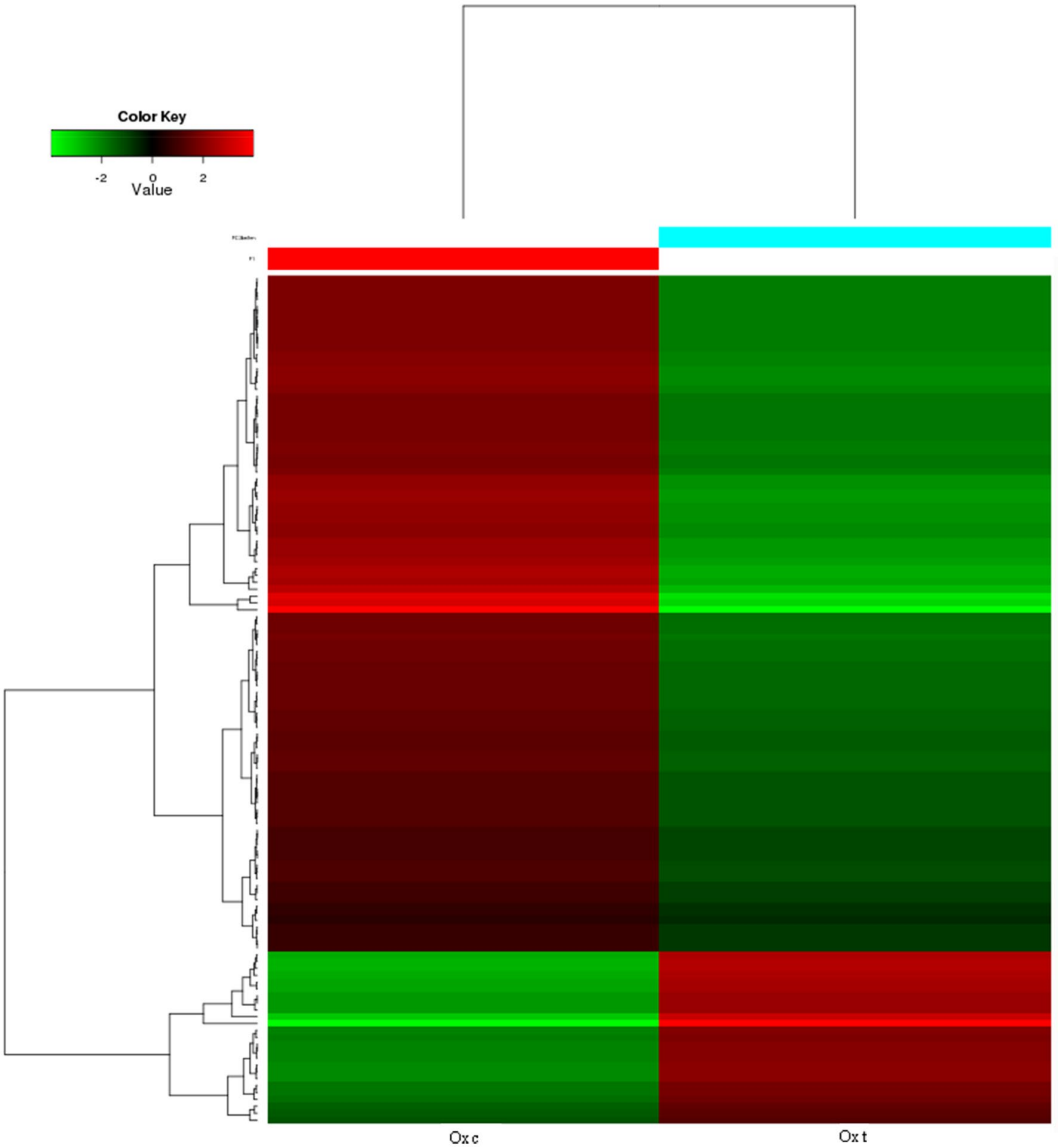
Hierarchical clustering of differentially expressed genes during early regeneration (fold change > 2 and FDR < 0.05). Red indicates up-regulated expression, whereas green indicates down-regulated expression. Ox c: control group, red bar; Ox t: regeneration group (1 day post amputation), blue bar.

To identify the differentially expressed genes (DEGs) involved in regeneration processes, gene expression in the treated (1 day post amputation) to the control group was compared and selected using a statistical cutoff of fold change > 2 and FDR < 0.05. A hierarchical clustering heatmap was generated to represent the up- and down-regulated genes. A total of 243 genes could be detected; 50 were upregulated and 193 were downregulated (Fig. 6). Among them, 15 significantly up-regulated genes were annotated as *neurotrypsin, nitric oxide synthase 2* (*Nos2*), *deleted in malignant brain tumors 1* (*DMBT1*), *SCO spondin*, *endotubin*, *18S* protein and *28S* protein, which suggest these genes might be important during early regeneration and thus candidates for further research (Table 3). Numerous of unknown genes were also found to have significantly different expression levels, which indicated that the process of regeneration might involve some new genes. Besides, lack of well-annotated genomes/transcriptome or technical artifacts would also increase the number of new genes.

**Table 3:** List of significantly up-regulated genes (fold change > 2 and FDR < 0.05) in early regeneration of *O. ximen*.

GeneID	Annotation	logFC	*p* Value	FDR
TRINITY_DN51273_c0_g1	Deleted in malignant brain tumors1 (DMBT1)	3.037675	4.57E−05	0.015983
TRINITY_DN50786_c0_g1	Neurotrypsin	3.1618	9.29E−06	0.003945
TRINITY_DN55189_c1_g3	Nitric oxide synthase 2 (Nos2)	3.184915	8.04E−06	0.003433
TRINITY_DN43190_c0_g1	Monocarboxylate transporter 12	3.20869	8.96E−05	0.028707
TRINITY_DN51913_c0_g1	SCO spondin like	3.270135	5.18E−06	0.002397
TRINITY_DN50786_c0_g2	Apical endosomal glycoprotein	3.393203	2.68E−06	0.001388
TRINITY_DN55867_c1_g3	ncRNA protein	3.454196	2.06E−05	0.007981
TRINITY_DN46494_c2_g1	28S protein	4.584054	3.50E−07	0.000258
TRINITY_DN44012_c2_ g2	28S protein	4.934653	3.60E−07	0.00026
TRINITY_DN44852_c0_g1	18S protein	5.806989	1.86E−09	2.93E−06
TRINITY_DN56797_c1_g1	18S protein	5.822492	1.69E−09	2.73E−06
TRINITY_DN55100_c4_g4	28S large subunit protein	6.03928	1.63E−07	0.000133
TRINITY_DN55100_c4_g6	28S protein	7.551671	0.000126	0.037895
TRINITY_DN46867_c0_g1	rRNA promoter binding protein	8.690415	4.00E−07	0.000278
TRINITY_DN44852_c0_g2	18S protein	9.049187	5.90E−08	5.49E−05

**Figure 7:**
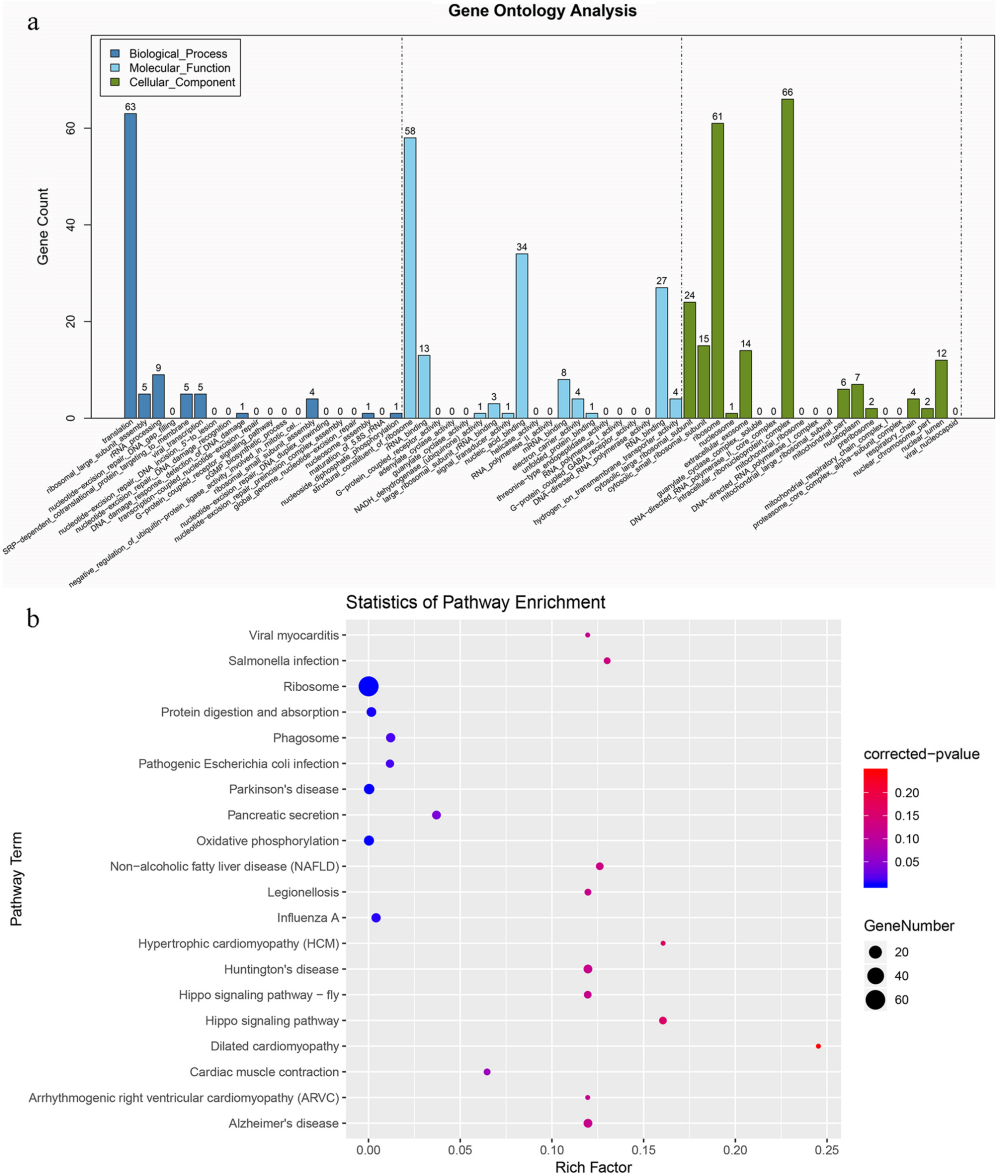
Analysis of differentially expressed genes between Ox t (1 day post amputation) and Ox c. (**a**) GO classification. The unigenes were classified into three main categories: molecular function (MF), cellular components (CC), and biological process (BP). (**b**) KEGG pathway enrichment analysis of the differentially expressed genes (www.kegg.jp/kegg/kegg1.html).

After the data analysis, the DEGs between the treated (1 day post amputation) and control groups were classified into 124 GO subcategories (78 subcategories for biological process, 26 subcategories for molecular function, and 20 subcategories for cellular component, *p* < 0.01). Translation, structural constituent of ribosome, ribosome and intracellular ribonucleoprotein complex subcategories contained the most DEGs and were treated as the focus of the analysis (Fig. 7). To further explore the mechanisms of regeneration, the DEGs were mapped to 132 KEGG pathways. DEGs were mainly involved in material metabolism and signal transduction, such as pathogenic *Escherichia coli* infection, arrhythmogenic right ventricular cardiomyopathy (ARVC), Salmonella infection, Huntington’s disease, Alzheimer’s disease, and Hippo signaling pathways were related to structure and signal transduction. The top 20 most abundant differentially expressed signaling pathways are listed (Fig. 7b).

### Candidate genes involved in regeneration processes

To better understand the molecular changes during early regeneration, the putative genes that have been implicated in other regeneration models were identified using BLAST searches (Supplementary file Table S4). A total of 130 transcripts were found in this study. Among these genes, *brachyury*, *cyclin B*, *Hox A2*, *Indian hedgehog*, *myc*, *notch1*, *notch4*, *PRDM8*, and *PRDM9* were up-regulated, while *notch2*, *neurogenin*, *matrix metalloproteinase*, and *glutamine synthetase* were detected to be down-regulated (|logFC|> 1). Only *cyclin B* showed significantly increased expression during early regeneration and no significantly down-regulated genes were detected (|logFC|> 1, FDR < 0.05).

### *Hox* genes and the preliminary expression pattern

**Figure 8:**
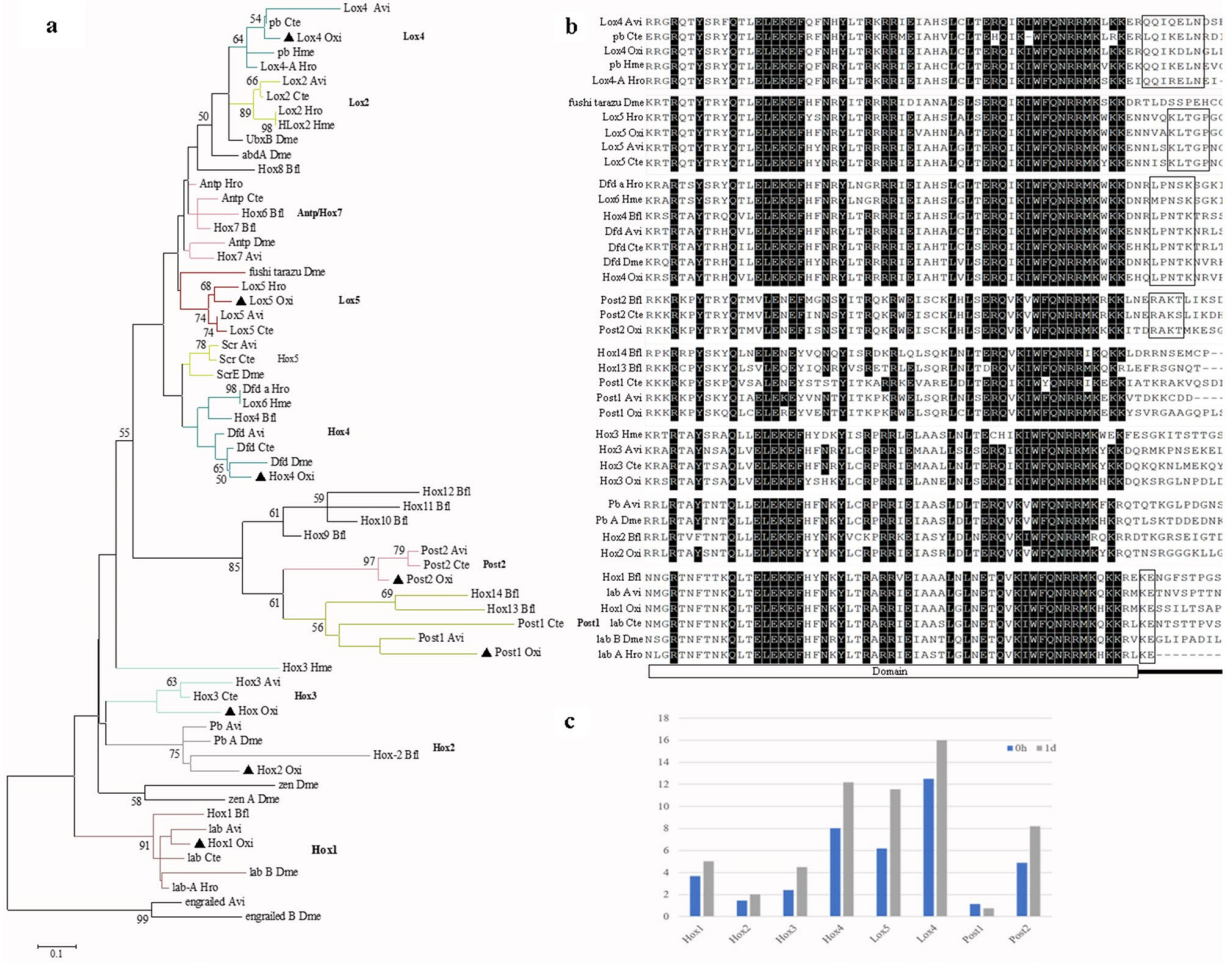
*Hox* genes of *O. xiamen*. (**a**) Phylogenetic tree of homeodomain genes. (**b**) Homeodomain sequence alignment. (**c**) Expression pattern of *Hox* genes in *O. xiamen* (0 h and 1 day post amputation). Residues with less than 80% of identity are not colored. Dashes represent missing data. Sequences marked with boxes contain the signatures found in flanking regions. The figures were created with MEGA 5.05. Avi: *Alitta virens*; Bfl: *Branchiostoma floridae*; Cte: *Capitella teleta*; Dme: *Drosophila melanogaster*; Hme: *Hirudo medicinalis*; Hro: *Helobdella robusta*; Oxi: *Ophryotrocha xiamen.*

In the phylogenetic analysis, 8 out of 43 homeodomain-like fragments were assigned to the anterior class *Hox* gene *Hox1*, *Hox2*; a *Hox3* gene; central class genes *Hox4*, *Lox5* and *Lox4*; and the posterior genes *Post1* and *Post2* (Fig. 8, Supplementary Fig. S4). No support for *Hox5* or *Lox2* orthologs was found. All genes except *Post1* were up-regulated in the early regeneration stage (|logFC|> 1, FDR < 0.05).

## Discussion

In the present study, we first describe *O. xiamen* **sp. nov.** collected from the Baicheng Bay of Xiamen. To establish a confirmed taxonomic position, the identity of this species is revised based on molecular tools combined with morphological characters^19,31^. Using morphological characters and phylogenetic analyses based on the mitochondrial gene *COI* and nuclear gene *H3*, we describe a new species, *Ophryotrocha xiamen*. Members of the genus *Ophryotrocha* are similar to each other in external morphology. For ‘labronica’ clade, all members are primarily gonochoristic and most have the diploid complement of chromosomes of 2n = 6 according to previous study^32^. This clade is distinguished from other species by their morphological features, such as rosette glands, jaw, and egg morphology^18,32,33^. In the phylogenetic tree, *O. xiamen* is positioned within the ‘labronica’ clade. The morphological characteristics also agree with this classification. The difference between *O. xiamen* and two closely related species is compared according to previous studies^32^. Maximum number of segments in *O. xiamen* is observed to be 24. While in *O. japonica* and *O. notoglandulata*, the maximum number of segments are 28 and 36, respectively. The median connection between eyes and long larval pygidial stylus exist in both *O. xiamen* and *O. notoglandulata*, but not in *O. japonica*. At larval release stage, 2–3 segments are observed in both *O. xiamen* and *O. japonica*. We observe a single rosette gland in the median position of posterior segments which mostly start from segment 12. The jaws, molting from the initial larval jaws to P1and P2 and finally the K-maxillae, develop at an earlier age in males than in females. In addition, females are observed to take care of their egg-cocoon until hatching and this phenomenon seems universal in *Ophryotrocha*. Within Annelida, mucus constitutes key factors in tube building, egg protection, and the prevention of proliferation of pathogenic microorganisms making it a particularly attractive class of biocidal agents^34^.

In some studies, it has already been advocated that new models that are amenable to molecular, cellular, and functional analyses are required to better understand the mechanisms of regeneration^35,36^. Based on their rapid individual growth rates and high regeneration efficiency, *O. xiamen* seemed to be a well-suited model to study the mechanisms of regeneration.

A rapid posterior regeneration process that followed a reproducible path and timeline allowed us to explore changes at different time points. Similar to *Platynereis dumerilii* (*P. dumerilii*)^15^, the whole process in *O. xiamen* was also subdivided into two continuous phases, regeneration per se (first 3 days) and post-regenerative growth. In annelids, epimorphosis is common mode of posterior regeneration and often involves the formation of a blastema comprised of an outer sheet of epithelial cells and an inner mass of mesenchyme-like cells that finally formed a complete posterior part. Our results showed that the amputated part could add new segments posteriorly and then grow to its original size with a similar growth rate even after multiple amputations. This phenomenon has been explained by the fact that the worm is able to ‘sense’ the site that has been cut and adjust its growth accordingly^15^.

No anterior segment regeneration was observed unless part of the prostomium remained intact. Through the release of hormones, the brain promoted or inhibited regeneration in some annelids^12,37^. It was previously reported that no further replacements occurred after K-type jaws formed in *Ophryotrocha*^1,38,39^. Thus, more details about the molecular mechanisms between posterior and anterior regeneration in *O. xiamen* is needed to better understand the essential foundation for future mechanistic and comparative studies of regeneration.

The regeneration process involves complex morphological changes, but only 243 transcripts with significantly different transcript levels were found in *O. xiamen*. It seemed that genes involved in the regeneration and regular growth process largely overlapped and had been demonstrated to have similar expression patterns^15,17,40^. On the other hand, lack of replication could also affect the detection of DEGs^41,42^. During regeneration processes, neurotrypsin and SCO-spondin were thought to be crucial for cognitive brain functions and they regulated the balance between neuroepithelial proliferation and differentiation, respectively^43,44^. Both DMBT1 and endotubin were required for enterocyte morphogenesis and differentiation^45,46^. DMBT1 was also known as a protein that functions in innate immunity, inflammation, and angiogenesis by influencing the proliferation, migration, and adhesion of endothelial cells^46,47^. A previous study showed that genes involved in the *Wnt/β-catenin* signaling pathway were crucial for early regeneration^48,49^.

In the early stages of regeneration, inflammation and apoptosis factors initiated the downstream process of development^50,51^. All of them were reported to play important roles in early embryonic development, involving several genes with integral roles in the re-specification of regenerated structures in several annelid species^2^. Due to the scarcity of annelids genomic data used for annotation, a large set of potentially novel DEGs in *O. xiamen* were identified. Previous studies revealed that unknown genes that showed similar expression trends with key regulators might be important and were required during regeneration processes. Thus, well annotated genome data of *O. xiamen*, which is already in progress, are needed to explain the exact gene functions and regeneration mechanisms.

To further understand the regeneration mechanisms in annelids, it will be of particular importance to identify genes that are specifically related to this process. By using BLAST searches, we found regeneration-related or putative genes that have been implicated in regeneration in other regeneration models^52^. All of these genes were reported to be involved in regeneration processes, including wound healing, blastema formation, cell proliferation control and morphogenesis^15,17,53^. Among these candidate genes, only *cyclin B* was significantly up-regulated which needed to be further confirmed. Several studies indicated that the function of cyclin B protein in invertebrates might have a dual role as *cyclin B1* and *cyclin B2* which was suggested to participate in the reorganization of different aspects of the cellular architecture at mitosis and have different functions in the cell cycle^54,55^. The early regeneration stage is a rapid process in annelids that consists of muscular contraction and epithelium formation^12^. Thus, *cyclin B* in *O. xiamen* was supposed to have the same functions in regulating the cell cycle during early regeneration.

Most of the *Hox* genes identified from *A. virens*, *P. dumerilii*, and *C. teleta* were detected in early blastema^13^. In the present study, 8 *Hox* genes were identified, but not *Hox5* or *Lox2*. In *P. dumerilii*^14^, *Hox5* and *Lox2* were also not to be expressed in the components of the regenerating nervous system or in the posterior growth zone. However, the expression of *Hox5* in *A. virens* was detected in the whole body and downregulation occurred at 10 h post amputation^10^. In this study, the absence of *Hox5* and *Lox2* in the transcriptome of both early regeneration and normal adult growth process of *O. xiamen* might be due to low transcription level during these stages. The down regulated *Post1* which was reported to be associated with the formation of new chaeta seemed to have a limited function in early regeneration^14^. Other homeodomain fragments clustered with the genes, such as *Cdx*, *even-skipped* and *engrailed*, were also identified (Supplementary Table S4). In addition to cellular-level studies, molecular-level studies are needed to provide a complete understanding of regeneration processes.

## Conclusions

In this study, the newly identified *O. xiamen* is suitable for studying the mechanism of regeneration. Comparative transcriptome analysis provides the expression changes during early regeneration in this species. Genes involved in regeneration, especially *Hox* genes, have also been investigated to reveal the similarity in regeneration mechanisms among related species. We hope that further investigations based on *O. xiamen* as a new model organism will provide deep insights into the regeneration process at the morphological and molecular levels and will stimulate interest in evolutionary research.

## Supplementary Information

Supplementary Information.
