# Supplementary material for: Morphological and molecular features of early regeneration in the marine annelid Ophryotrocha xiamen
Source: Sci Rep. 2022 Feb 2;12:1799. doi: 10.1038/s41598-022-04870-3 (PMC8810878; doi:10.1038/s41598-022-04870-3)

Supplementary files

Table S1. Rate of development for *O. xiamen* in mass culture at 25°C.

**Time (days)**	**Stage**
**0**	Spawning, 93±22μm;
**7**	Hatching, 2 segments, 294±23μm;
**8**	3 segments, 331±27μm;
**9-18**	4-12 segments, one new segment formed each day, 2770±207μm;
**19-26**	12-15 segments, changing to K-maxillae and oocytes were observed in the coelom, 4392±305μm;
**26-28**	First spawning, 16 segments,4806±854μm;

Supplementary files

Table S2. BLAST results for candidate regeneration genes.

Gene name	GeneID	logFC	PValue
AP 2	TRINITY_DN52415_c0_g1	-0.069457	0.923135
Brachyury	TRINITY_DN42461_c0_g1	-1.440205	0.058066
β-catenin	TRINITY_DN51665_c1_g1	-0.167305	0.810876
	TRINITY_DN55669_c0_g1	0.1429082	0.827414
Cyclin B	TRINITY_DN39941_c1_g1	-4.281088	2.97E-06
Elav 2	TRINITY_DN50942_c1_g1	-0.162993	0.804106
Elav 3	TRINITY_DN54482_c3_g1	-0.223634	0.737559
Engrailed 1	TRINITY_DN47607_c3_g1	-0.636758	0.368455
Even skipped	TRINITY_DN42760_c0_g2	0.8492827	0.290856
FGFR	TRINITY_DN52023_c0_g1	-0.263431	0.709297
FGFR 2	TRINITY_DN52179_c1_g1	-0.151391	0.830876
FGFR 3	TRINITY_DN56126_c0_g3	0.2137296	0.747497
Frizzled 1/2/7	TRINITY_DN46576_c4_g1	0.4440734	0.501784
	TRINITY_DN56587_c5_g2	0.0706987	0.916602
	TRINITY_DN52855_c4_g1	0.4562889	0.493083
	TRINITY_DN56183_c1_g3	0.1389833	0.861851
Frizzled 5/8	TRINITY_DN54863_c1_g1	-0.247489	0.718499
Glial cells missing	TRINITY_DN51440_c1_g2	0.1349317	0.841424
Glutamine synthetase	TRINITY_DN57172_c3_g1	0.0623375	0.932407
	TRINITY_DN50997_c0_g2	1.8488023	0.00624
	TRINITY_DN47546_c0_g2	-0.600189	0.383562
	TRINITY_DN57172_c1_g1	-0.272388	0.67874
	TRINITY_DN45467_c3_g1	-0.105373	0.872483
	TRINITY_DN49519_c6_g1	-0.069102	0.916144
Glutamine synthetase 1	TRINITY_DN42272_c0_g1	-0.868281	0.221258
Glutamine synthetase 2	TRINITY_DN49609_c5_g1	-0.146033	0.827136
Hedgehog	TRINITY_DN48827_c1_g1	-0.60647	0.355374
	TRINITY_DN50879_c1_g1	0.1882405	0.791589
Hox 1	TRINITY_DN47787_c3_g1	-0.323216	0.644424

Hox A2	TRINITY_DN47787_c2_g2	-5.656062	0.056522
	TRINITY_DN53831_c2_g1	0.3130237	0.660865
Hox D3	TRINITY_DN44426_c0_g1	-0.096636	0.9053
Hox B3	TRINITY_DN44749_c1_g2	0.822631	0.241178
Hox A5	TRINITY_DN43052_c0_g1	-0.652432	0.405843
Hox A7	TRINITY_DN45034_c0_g1	0.1177488	0.862513
Hunchback 6	TRINITY_DN57053_c0_g1	-0.416241	0.534594
Indian hedgehog	TRINITY_DN51244_c0_g3	-1.286844	0.05332
	TRINITY_DN44501_c0_g1	0.1115953	0.864936
jnk	TRINITY_DN50322_c3_g1	0.0125106	0.985919
	TRINITY_DN52667_c2_g1	-0.618179	0.380556
Lox5	TRINITY_DN47787_c4_g1	0.7368751	0.276002
	TRINITY_DN55293_c0_g1	-0.082558	1
Matrix metalloproteinase	TRINITY_DN47337_c2_g2	-0.395911	0.567616
	TRINITY_DN46009_c2_g1	1.4156524	0.056243
	TRINITY_DN56019_c0_g1	0.2554083	0.707887
Myc protein	TRINITY_DN42804_c0_g1	-1.027034	0.125816
Nanos 2	TRINITY_DN43081_c0_g1	0.1740114	0.806964
Neurogenin	TRINITY_DN41201_c0_g1	1.8552382	0.036253
Noggin 3	TRINITY_DN50135_c3_g2	0.2790288	0.699449
Notch 1	TRINITY_DN58451_c0_g1	-4.684393	0.285714
	TRINITY_DN20020_c0_g1	-1.527566	0.107412
	TRINITY_DN29246_c0_g1	-0.07831	1
	TRINITY_DN54313_c3_g1	-0.236146	0.719236
	TRINITY_DN55225_c0_g1	-0.083108	0.923432
	TRINITY_DN48117_c3_g1	0.0149521	1
	TRINITY_DN60732_c0_g1	-4.684393	0.285714
Notch 2	TRINITY_DN47881_c0_g1	-0.443386	0.520091
	TRINITY_DN45998_c0_g1	-0.135907	0.837234
	TRINITY_DN56432_c0_g1	-0.145319	0.824784
	TRINITY_DN55521_c2_g1	0.0433318	0.947385
	TRINITY_DN52638_c0_g1	-0.129352	0.846949
	TRINITY_DN55521_c0_g2	0.3202593	1
	TRINITY_DN44813_c1_g1	5.004652	0.161491

		1	
Notch 3	TRINITY_DN54698_c0_g1	-0.547887	0.410621
Notch 4	TRINITY_DN55116_c0_g1	-1.550856	0.658385
OTX2	TRINITY_DN54946_c3_g2	-0.098026	0.895996
Patched 1	TRINITY_DN52517_c0_g1	-0.043186	0.948565
Paired box protein 1	TRINITY_DN47080_c0_g1	-0.294107	0.688856
Paired box protein 2/5/8	TRINITY_DN55811_c2_g1	-0.025203	0.970902
	TRINITY_DN54328_c1_g1	0.1536783	0.833855
	TRINITY_DN53196_c1_g2	-0.376722	0.647858
Paired box protein 6	TRINITY_DN55465_c0_g2	-0.333257	0.611164
Paired box protein 7	TRINITY_DN48786_c0_g1	-0.454189	0.554538
Piwi 1	TRINITY_DN40503_c0_g5	0.1778022	0.884696
	TRINITY_DN40503_c0_g2	-0.883495	0.348276
	TRINITY_DN50972_c4_g1	-0.37897	0.566488
	TRINITY_DN53970_c1_g2	-0.129658	0.853506
	TRINITY_DN48835_c4_g2	-0.209217	0.761163
	TRINITY_DN47628_c0_g1	-0.136714	0.849178
	TRINITY_DN55620_c3_g1	0.052939	0.952359
	TRINITY_DN52339_c2_g4	-0.571433	0.386026
PL10	TRINITY_DN50803_c1_g1	-0.005997	0.992991
Post1	TRINITY_DN50156_c0_g2	-0.774045	0.545915
Post2	TRINITY_DN54129_c2_g4	0.5837933	0.392654
PRDM1	TRINITY_DN53983_c0_g2	-0.658303	0.320059
	TRINITY_DN47608_c2_g1	-0.957907	0.157734
PRDM8	TRINITY_DN37984_c0_g1	-0.942911	0.479207
	TRINITY_DN55718_c3_g3	-4.127215	0.52381
	TRINITY_DN37984_c0_g2	0.1778022	0.884696
PRDM9	TRINITY_DN36494_c0_g1	-2.930392	0.084472
	TRINITY_DN30529_c0_g1	1.4649996	0.251568
	TRINITY_DN36740_c0_g2	-0.081174	1
	TRINITY_DN54945_c3_g1	-0.083075	0.938492
	TRINITY_DN48110_c4_g1	-0.459188	0.573359
	TRINITY_DN44310_c4_g1	0.4346534	0.590644
	TRINITY_DN46027_c3_g1	0.2690475	0.699656
PRDM 12	TRINITY_DN53357_c2_g2	-0.174901	1
	TRINITY_DN53357_c2_g3	0.7530112	0.289684
PRDM14	TRINITY_DN47236_c0_g1	-0.081744	1

PRDM15	TRINITY_DN54089_c2_g1	-0.119301	0.856341
Proliferating cell nuclear antigen	TRINITY_DN36152_c0_g1	0.428577<br>1	0.519143
Regeneration-upregulated protein 5	TRINITY_DN54886_c1_g1	-0.242962	0.724817
	TRINITY_DN48384_c0_g1	-0.867537	0.204674
Secreted frizzled related protein 3	TRINITY_DN48453_c3_g1	0.286532<br>8	0.668752
Secreted frizzled related protein 5	TRINITY_DN45668_c0_g1	-0.397611	0.547967
Slit	TRINITY_DN55960_c1_g1	-0.574063	0.386043
TDRD 1	TRINITY_DN55813_c1_g2	-0.462536	0.485105
	TRINITY_DN54305_c0_g1	-0.014749	0.983729
	TRINITY_DN46550_c0_g2	-0.724414	0.273781
	TRINITY_DN43045_c0_g1	-0.511757	0.501624
TDRD 3	TRINITY_DN47033_c0_g2	-0.450558	0.502347
	TRINITY_DN47033_c0_g1	-0.470426	0.476506
TDRD 5	TRINITY_DN55362_c1_g1	0.084574	0.916452
TDRD 6	TRINITY_DN52571_c0_g2	-0.001798	1
	TRINITY_DN46804_c4_g3	-0.288791	0.740515
TDRD 12	TRINITY_DN55984_c0_g1	-0.057999	0.9396
Unc 119	TRINITY_DN53045_c1_g1	0.344893<br>2	0.601959
Vasa	TRINITY_DN49109_c0_g1	-0.102869	0.876775
Wnt1	TRINITY_DN50831_c1_g1	-0.136537	0.842625
	TRINITY_DN44527_c3_g1	-0.228078	0.744683
Wnt2	TRINITY_DN42833_c0_g1	-0.593314	0.544247
	TRINITY_DN444_c0_g1	-0.394086	1
Wnt 4	TRINITY_DN52705_c0_g1	-0.327043	0.661501
Wnt5	TRINITY_DN56566_c3_g1	-0.511991	0.448146
Wnt6	TRINITY_DN11919_c0_g1	0.031954<br>6	1
Wnt 7	TRINITY_DN50386_c3_g1	0.136879<br>4	0.841783
Wnt 9	TRINITY_DN47835_c1_g1	0.832629<br>3	0.243201
Wnt 10	TRINITY_DN44179_c0_g2	-0.631054	0.42641
Wnt11	TRINITY_DN53345_c4_g1	-0.151275	0.830341
Wnt 16	TRINITY_DN49492_c3_g1	-0.453609	0.521155

Supplementary files

Table S3. Genbank accession numbers for *Ophryotrocha* taxa examined.

Taxon	H3	COI
Exallopus jumarsi	—	JQ310755
Iphitime hartmanae	GQ415491	GQ415472
Ophryotrocha adherens	JQ310768	JQ310756
O. alborana	GQ415492	GQ415473
O. costlowi	JQ310770	JQ310757
O. craigsmithi	GQ415493	GQ415474
O. diadema	JQ310771	JQ310758
O. eutrophila	GQ415494	GQ415475
O. flabella sp. nov.	JQ310772	JQ310759
O. geryonicola	GQ415495	GQ415476
O. globopalpata	GQ415496	GQ415477
O. gracilis	GQ415497	EF464545
O. hartmanni	JQ310773	EF464546
O. japonica	GQ415498	GQ415478
O. labronica	GQ415499	GQ415479
O. langstrumpae	JQ310774	JQ310760
O. lobifera	GQ415500	GQ415481
O. longicollaris sp. nov.	JQ310775	JQ310761
O. longidentata	GQ415501	GQ415482
O. macrovifera	JQ310776	JQ310762
O. maculata	JQ310777	GQ415483
O. magnadentata sp. nov.	JQ310778	JQ310763
O. nauarchus sp. nov.	JQ310779	JQ310764
O. notoglandulata	JQ310780	EF464542
O. permanae	GQ415502	GQ415484
O. puerilis	GQ415503	GQ415485
O. robusta	JQ310781	EF464547
O. rubra	GQ415505	GQ415487
O. scutellus	GQ415506	GQ415488
O. shieldsi	JQ310782	HM181931
O. socialis	JQ310783	JQ310765
O. vivipara	—	JQ310766
O. xiamen	MT551185	MT561413
O. sp. nov.	KP731549	EF464538
O. sp. sanya sp.2	EF464537	—
O. sp. Qingdao	EF464539	—
O. Jiaolongi	MF398972	KY906965
O. orensanzi	KC123180	KX398990
Dorvillea similis	—	DQ317857
Dorvillea albomaculata	JQ310784	EF464550

Eunice pennata	DQ779731	AY838870
Parougia bermudensis	JQ310785	JQ310767
Parougia eliasoni	GQ415507	GQ415489

Supplementary files

Table S4. Gene accession numbers or transcriptome data of *Hox* genes used in this analysis.

	**Avi**	**Cte**	**Hme**	**Hro**	**Bfl**	**Dme**	**Oxi**
Hox1	AAD46166.2	ABY67952.1		XP_009014546	BAA78620.2	AFH06272.1	TRINITY_DN47787_c3_g1
Hox2	AAD46167.2	ABY67953.1	AAB35067.1		CAA84515.1	AAF54089.3	TRINITY_DN53831_c2_g1
Hox3	AAD46168.1	ABY67954.1	AAB35372.1			AAF54087.1; AAF54088.1	TRINITY_DN44749_c1_g2
Hox4	AAD46169.2	ABY67955.1	AAB82458.1	AAB61441.1	BAA78622.1	AAF54083.2	TRINITY_DN43115_c0_g1
Hox5	AAD46170.1	ABY67956.1				AAS65104.2	
Lox5	AAD46174.2	ABY67957.1		AAB61442.1	ABX39490.1	AAF54081.1	TRINITY_DN47787_c4_g1
Antp	ABD04657.1	ABY67962.1		009028115.1	ABX39491.1	AAA70216.1	
Lox4	AAD46172.1			XP_009010732.1	ABX39492.1		TRINITY_DN45034_c0_g1
Lox2	AAD46171.2	ABY67959.1	sp|P21523.1|	CAA78665.1	ABX39493.1	AAF55356.1; AAF55360.2	
Post1	AAD46175.1	ABY67961.1			CAA84522.1		TRINITY_DN50156_c0_g2
Post2	AAD46176.2	ABY67960.1			AAF81909.1		TRINITY_DN54129_c2_g4
Engrailed	ABD04655.1				AAF81903.1	NP_725059.1	

Supplementary files

Figure S1. Egg tube of *O. xiamen* with large number of larvae ready to be released, female on surfaceof the tube.

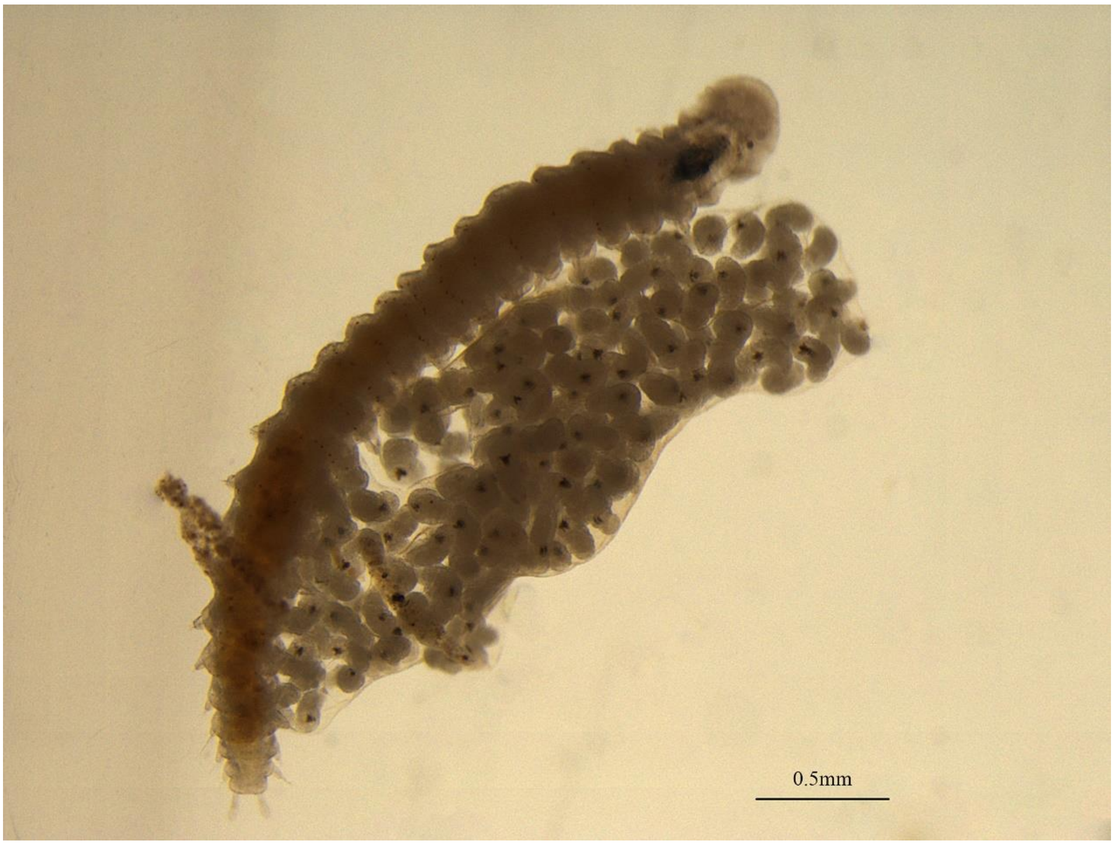

Supplementary files

Figure S2. Anterior regeneration in *O. xiamen*. The morphology changes were observed in twoweeks after amputation and kept till death. The white arrows showed the amputation sites. Scalebars were showed in the bottom of each image.

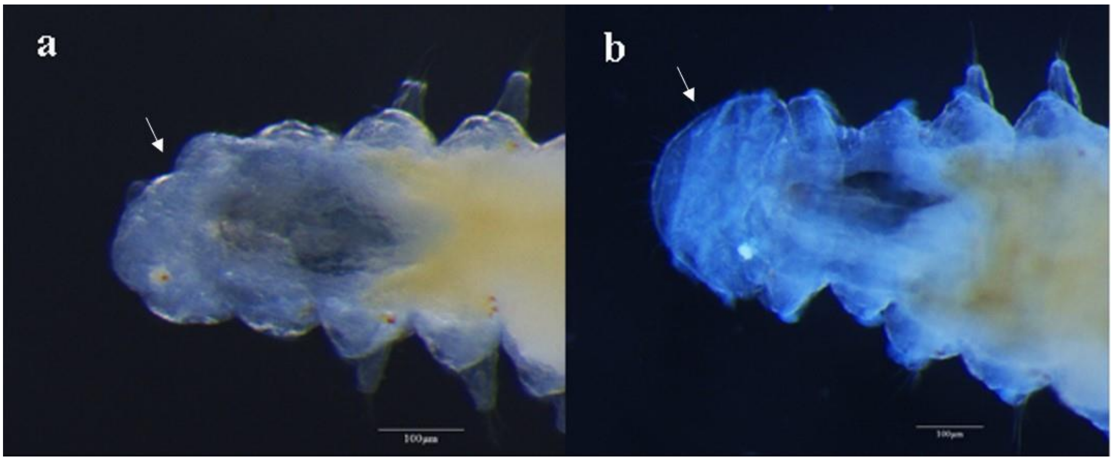

Supplementary files

Figure S3. Survival of posterior and anterior amputees with different position of amputation plane(post-pharynx segments 0, 2-4, 6-8, 10-12). Each group (n=15) were performed in triplicate.Survival rates were calculated as the means±SD. ap: anterior amputees; pp: posterior amputees.

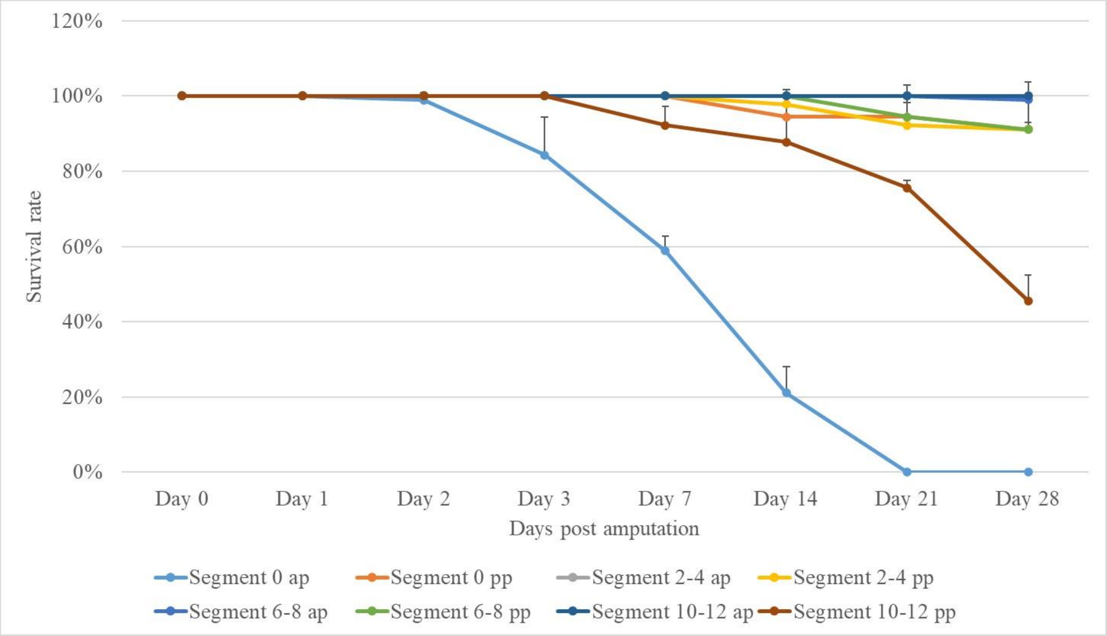

Supplementary files

Figure S4. Phylogenetic analysis using 43 homeodomain-like fragments. Oxi: *Ophryotrocha xiamen*. The figures were created with MEGA 5.05.

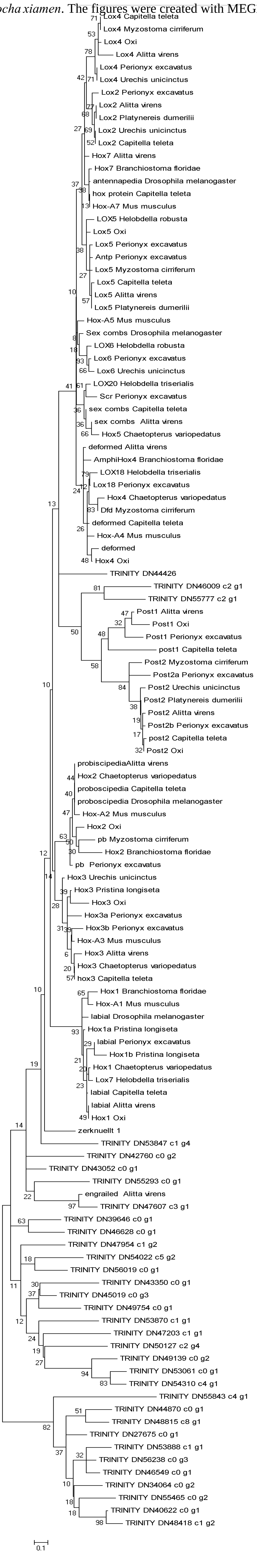

Supplement: Supplementary file 1 — Supplementary Information. [file 41598_2022_4870_MOESM1_ESM.pdf]
